# Could Blood Transfusion Increase the Risk of Alzheimer’s Disease? A Narrative Review

**DOI:** 10.3390/healthcare13050452

**Published:** 2025-02-20

**Authors:** Xiaoyue Li, Renjun Pei, Zhangcheng Fei, Zhongsheng Chen, Fangzhao Lin, Pan Sun, Haijun Cao

**Affiliations:** Institute of Blood Transfusion, Chinese Academy of Medical Sciences and Peking Union Medical College, Chengdu 610052, China; lixiaoyue@pumc.edu.cn (X.L.); peirj0803@163.com (R.P.); zhangchengfei94@tmmu.edu.cn (Z.F.); 15321116371@163.com (Z.C.); fangzhao.lin@ibt.pumc.edu.cn (F.L.)

**Keywords:** Alzheimer’s disease, blood transfusion, amyloid-β, tau, adverse events, blood management

## Abstract

Alzheimer’s disease (AD) is the most common progressive neurodegenerative disease, and its pathogenesis is complex. In addition to amyloid-β and phosphorylated tau, inflammation and microbial infections also play a role in the development of AD. Currently, there is no effective clinical intervention to cure AD or completely halt its progression. Blood transfusion, a critical life-saving medical procedure widely employed in modern healthcare, faces growing demand due to global population aging. However, whether blood transfusion could increase the risk of AD is still not clear. Aβ and tau play major roles in the pathogenesis of AD and may possess the potential for transmission through blood transfusion. Iron overload and chronic inflammation, which can independently influence AD pathogenesis, may result from repeated transfusions. Additionally, herpesvirus, known to accelerate AD progression, can also be potentially transmitted by blood transfusion. In this study, recent advances in the associations between blood transfusion and the occurrence and development of AD were reviewed, and whether blood transfusion could increase the risk of AD was discussed. Furthermore, the related proposals for blood management and future research were advanced to provide references for the prevention and control of AD.

## 1. Introduction

Alzheimer’s disease (AD) is the most common cause of dementia, with characteristic brain changes including the accumulation of the abnormal proteins amyloid-β (Aβ) and phosphorylated tau, as well as the damage and destruction of neurons [1]. With the aging of the global population, the prevalence of AD is increasing year by year [2]. It is estimated that the number of patients with AD and other dementias will reach 152.8 million in 2050, which will cause a huge burden on public expenditure [3]. There are multi-level mechanisms contributing to AD, ranging from age, genetics, and life exposure to molecular abnormalities and brain network impairment [4]. Currently, there is a certain understanding of the pathogenesis of AD, but no effective means for the prevention and treatment of AD.

Blood transfusion is an important measure of clinical treatment, which plays an irreplaceable role in the treatment of some diseases. However, the blood itself is the carrier of pathogens and foreign substances. Accordingly, there is a risk of transmitting diseases caused by blood transfusion. In the context of global aging, the number of AD patients and blood demand will continue to increase. Whether blood transfusion can increase the risk of AD should be an urgent concern. Herein, the studies on the association of blood transfusion with AD were summarized, and related suggestions were put forward to provide some references for the prevention and control of AD.

## 2. Methods

The eligible English or Chinese literature published before August 2024 was retrieved from PubMed, Embase, Cochrane Library, ClinicalTrials.gov, China National Knowledge Infrastructure, and Wanfang Database. Simultaneously, the citation tracing method was employed to retrieve relevant references and explore the other related literature. The literature retrieval was conducted by combining subject words and free words. The search terms were Alzheimer*, cognitive dysfunction, cognitive impairment, dementia, transfusion, transmission, contagious, etc. A preliminary screening was conducted on the titles and abstracts of the literature. The full texts were obtained for further analysis and article organization.

## 3. Results

### 3.1. Main Hypotheses of AD

The difficulty in preventing and treating AD is related to the lack of a clear understanding of its pathogenesis. With the deepening of the study of AD, multiple hypotheses have been proposed from different viewpoints to elucidate its etiology. One of the most widely accepted hypotheses is the amyloid hypothesis, which holds that the overproduction, aggregation, and decreased clearance of Aβ are the primary factors in the pathology of AD [5,6,7]. Under normal conditions, the brain employs various pathways for Aβ clearance [8]. When the production and clearance of Aβ are out of balance, Aβ is deposited and triggers a cascade of events that causes neuronal damage and death, manifesting as progressive clinical dementia [9]. Moreover, it has been shown that the deposition of Aβ in the brain initiates AD pathogenesis, leading to the subsequent phosphorylation of tau, neuronal loss and dysfunction, as well as cognitive decline in numerous studies [7,10,11,12].

As another important pathophysiological feature of AD, neurofilament tangles are formed by the intracellular accumulation of hyperphosphorylated tau [13]. Tau is well-established as a microtubule-associated protein that plays an important role in maintaining the stability of microtubules and regulating microtubule assembly [14]. The tau hypothesis supposes that cognitive decline and neurodegeneration in AD are primarily driven by the onset and spread of tau pathology [15]. Much evidence indicates that the pathological state of tau is closely related to the progression of cognitive impairment in patients with AD [16,17,18].

Additionally, chronic inflammation in the brain is not only the main physiological mechanism of Aβ production, deposition, and senile plaque formation, but also a key factor of tau hyperphosphorylation, neurofibrillary tangles (NFTs), and neuronal degeneration [19]. Microbial infection, especially herpesvirus infection, also plays a promoting role in the pathology of AD. The antimicrobial hypothesis suggests that certain pathogens, such as herpesvirus, may induce Aβ deposition and thereby initiate the amyloid cascade, promoting the pathogenesis of AD [20]. In addition, other hypotheses are used to explain the pathogenesis of AD, such as the oxidative stress hypothesis [21], vascular hypothesis [22], etc. These hypotheses are not unrelated, but intertwined and complementary.

### 3.2. Association Between Blood Transfusion and AD

#### 3.2.1. Aβ

Aβ is identified as the primary constituent of meningovascular and cerebral parenchymal amyloid plaques, and it is produced by the sequential cleavage of β-amyloid precursor protein (APP) by β-secretase and γ-secretase [23]. It is prone to aggregate into conformations in the form of oligomers, protofibrils, and fibrils, which are detectable in the AD brain. Its deposition in the brain is often accompanied by synaptic damage and neuronal death.

It is known that a variety of nervous system diseases are characterized by pathological changes in a specific protein, such as Creutzfeldt–Jakob disease, which is caused by prion proteins with abnormal exogenous conformation [24,25,26]. There are some commonalities between Aβ and prions. Firstly, infectious prions and Aβ seeds exist in a wide range of sizes. For prions, the most potent are small and soluble. Similarly, Aβ seeds can be small oligomers or large fibrils, while oligomers are more neurotoxic [27]. Secondly, like prions, Aβ folds into strain-like variants both in vitro and in vivo [28,29]. These misfolded Aβ variants have significant differences in plaque morphology, conformational stability, and other biophysical characteristics [30]. Thirdly, Aβ and prions are durable, perhaps due to their highly stable state [31]. Finally, Aβ fibrillization can be “seeded” in a prion-like manner, in that pre-aggregated misfolded β-sheet can efficiently induce monomeric Aβ to obtain β-sheet and assemble into amyloid [32]. A wealth of evidence argues that Aβ leads to prion-like spreading throughout the brain during AD pathogenesis [33,34]. This provides an inference that Aβ may be transmitted from person to person like prions.

The seeding and spread of Aβ pathologies have long been studied in preclinical models as well as in human scenarios (Table 1). Preclinical studies have demonstrated that human Aβ can induce AD pathology in various AD mouse models through intracerebral or intravenous administration of brain homogenates, bone marrow cell transplantation, parabiosis models, and even blood transfusion. Simultaneously, there are documented case reports and theoretical inferences concerning the potential iatrogenic transmission of AD in human scenarios through the use of contaminated dura mater, cadaveric-derived human growth hormone (c-hGH), and surgical instruments harboring Aβ seeds.

- Preclinical models

Aβ can be transferred from the donor to the host through exogenous seeding, a phenomenon that may occur not only in transgenic mice but also in primates. The acceleration of Aβ deposition in the brain was observed following the intralingual injection of Aβ-rich brain extracts into Tg2576 mice [35]. This finding suggests that Aβ seeds can be transported from peripheral compartments to the brain via retrograde axonal transport, given that the tongue is a highly innervated organ [35]. The brain homogenate containing a large amount of Aβ was extracted from AD patients or amyloid precursor protein (APP) transgenic mice and then injected into the hippocampus of young mice [36]. Four months later, both sources of extractions caused Aβ deposition and cerebral amyloid angiopathy (CAA) in their brains, although rodent Aβ did not readily form amyloid. It was also found that Aβ deposits in the brains of young healthy rhesus monkeys could be induced by a homogenate containing Aβ from humans and marmosets [37]. It is not clarified if Aβ physically alters the structures of the normal proteins it encounters in the above circumstances. However, these provide a conjecture of the interpersonal transmission of AD since Aβ has prion-like activity and could act as a seed to induce Aβ pathology in the brain [38,43,52].

Exogenous Aβ might also enter the brain through the circulatory system and cause AD pathology. A single intravenous injection of Aβ seeds derived from human AD brain extracts into amyloid precursor protein/presenilin 1(APP/PS1) mice resulted in cerebral amyloid angiopathy within 180 days after injection [41]. Three-month-old wild-type mice were irradiated and then transplanted with bone marrow cells expressing human Aβ from APP/PS1 transgenic mice. Twelve months after transplantation, phenotypes such as Aβ plaques, neuronal degeneration, and behavioral deficits were observed in wild-type mice [39]. Meanwhile, the bone marrow stem cells from 12-month-old Tg2576 mice were injected via the tail vein into sex-matched 6- to 8-week-old APP-knockout mice. After 6–9 months, the rapid development of AD pathological hallmarks such as compromised blood–brain barrier integrity, elevated brain-associated Aβ levels, and cognitive impairment were observed [40]. The pathology of AD can be caused by Aβ produced outside the central nervous system, and Aβ in the blood can lead to the occurrence of AD.

**Figure 1:**
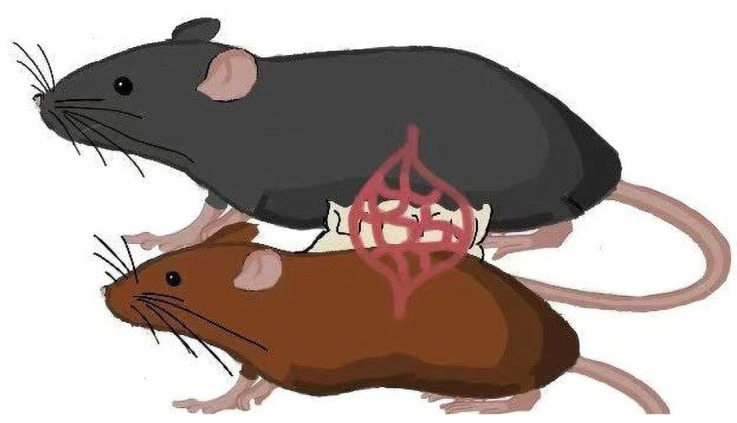
Surgery was performed to set up parabiosis between AβPP transgenic and wild-type mice.

In 2015, a parabiosis model of mice was used to connect AD mice carrying the Aβ mutant gene with their wild-type littermates by surgical suture so that they shared the same blood circulation (Figure 1) [38]. After 12 months, it was detected that the human Aβ produced by transgenic mice entered the blood circulation, accumulated, and produced vascular amyloid lesions in the brain of the wild-type mice. Moreover, there were other AD-related pathologies in the brains of the wild-type mice, including tau protein hyperphosphorylation, neuronal degeneration, neuroinflammation, hippocampal dysfunction, and so on. In 2023, Kunming mice aged 6–7 months were continuously injected with blood from 6-month-old APP/PS1 mice for 4 weeks [53]. Although previous behavioral studies had not found any changes in the cognitive function of Kunming mice, immunohistochemistry revealed an increase in the proportion of Aβ_42_, IBA-1, and GFAP-positive areas in the hippocampus after 1 month of injection, while the NeuN protein content decreased [54]. The infusion of blood from APP/PS1 mice may cause the deposition of Aβ_42_ in the brain, promote the activation of astrocytes and microglia, and lead to neuronal loss. Furthermore, young Tg2576 mice developed higher levels of brain amyloidosis after being infused with whole blood or plasma from old mice with extensive Aβ deposits in their brains, and the acceleration of brain Aβ deposition was reproducible in APP/PS1 mice [42]. These studies demonstrate that blood-derived Aβ can enter the brain, induce pathology related to Aβ, and cause neuronal functional defects and the occurrence of AD. According to the above outcomes, it can be inferred that in mouse models, Aβ can be transmitted to another individual through blood, thus inducing AD pathology.

- Human scenarios

Medical procedures such as growth hormone therapy and dural grafting are potential risk factors for the interpersonal transmission of Aβ [48,49,55]. Alzheimer-type gray matter and vascular Aβ pathology were found in the brain parenchyma and cerebral vessels of many young autopsies of patients with Creutzfeldt–Jakob disease who underwent dural grafting [44]. The Aβ deposition found in the autopsy was affected by dural grafting, and the Aβ deposition was triggered by the graft infecting the host blood [44]. The human c-hGH was administered to individuals with short stature, and approximately three decades later, Aβ deposition was observed in their brains [45]. Notably, many of these patients were relatively young and did not possess known AD-related risk factors. The biochemical assay of the injected c-hGH showed that it carried Aβ and tau [46]. The extracts could also induce amyloid deposition and CAA pathology when injected intracerebrally into AβPP transgenic mice [46]. In 2024, case reports emerged of five individuals who had received c-hGH contaminated with Aβ seeds during their childhood and exhibited signs of early-onset dementia and progressive cognitive impairment more than 30 years later, meeting the diagnostic criteria for AD [47]. We speculate that the Aβ in c-hGH enters the circulatory system by injection, then enters the brain, and initiates or promotes Aβ deposition in the brain.

In addition, Aβ proteopathic seeds, similar to prions, may be transmissible through surgical instruments or blood transfusions. Four patients who did not carry pathogenic mutations associated with early Aβ pathological development underwent neurosurgical procedures during their childhood or teenage years. However, they developed severe CAA about 30 years after the surgery, raising the possibility that Aβ pathology could be transmitted via surgical instruments carrying misfolded Aβ protein [50]. In 2023, Zhao et al. conducted a retrospective cohort study under the explicit assumption that donors with two or more intracerebral hemorrhages (ICHs) were likely to have CAA (an Aβ-driven disease) [51]. As a result, this study showed that individuals who received red blood cell transfusions from donors who subsequently experienced multiple spontaneous ICHs faced a considerably higher risk of developing spontaneous ICH [51]. Thus, the possibility of Aβ transmission via surgical instruments and blood transfusion should be taken into account [56].

#### 3.2.2. Tau

Tau is a multifunctional protein related to microtubule function. The stability of the microtubule structure in neurons is conducive to transmitting nutrients or information molecules between synapses. Tau was separated from the microtubules, adhered and gathered into paired helical filaments, and reassembled into NFTs in patients with AD. The main component of NFTs is hyperphosphorylated tau, which can destroy the structure of neurons, cause abnormal communication between neurons, and lead to neuronal apoptosis.

There is mounting evidence that hyperphosphorylated tau has the ability to self-propagate and spread. After human tau was injected into the brain of transgenic mice expressing Aβ, more endogenous tau was accumulated in the brain area near the injection site [57]. It was suggested that exogenous tau could promote the production and accumulation of endogenous tau in the animal brain in a way similar to contact transmission. Tau pathology just involves the neighboring regions in the absence of Aβ, so the presence of Aβ in the brain could accelerate the pathology of tau. Both human and animal studies argue that pathologically misfolded Aβ initiates the formation of prion-like tau in AD [58,59].

To test whether tau was also contagious in the human brain, Prusiner et al. developed sensitive cellular assays to quantify the self-propagating conformational forms of Aβ and phosphorylated tau in the brain [34]. After examining more than a hundred human brain specimens, they found that there were prion-like Aβ and tau proteins in the brains of patients with AD. The nerve fiber tangles formed by pathological tau were not randomly distributed in the brain. The fibers first gathered in the transentorhinal cortex and then spread to the adjacent brain parenchyma following a stereotypical spatiotemporal progression, which suggested that the tau itself or the pathological process that caused tau phosphorylation might be transmitted directly between cells through anatomical structures [60,61]. Based on the insights gained from autopsy studies, recently introduced molecular tau imaging methods have validated the spatiotemporal dissemination of pathological tau through the use of tau-specific ligands in positron-emission tomography and functional magnetic resonance imaging [62]. These techniques have shown that nodes with strong connections exhibit more tau pathology in AD, regardless of the intrinsic connectivity networks [62]. JW Vogel et al. used an epidemic spreading model to simulate the spread of tau [63]. Exogenous tau was placed in the entorhinal cortex of the brain and then its diffusion was simulated through measured functional and anatomical connections in this model. This diffusion pattern was compared with the actual model obtained by scanning multiple AD patients with tau-PET. It was found that the two models were very similar after calculation and analysis, which meant that tau in the brain could spread between brain cells through neuronal connections, and when Aβ existed, the transmission process was accelerated.

The level of tau in the plasma of patients with AD is significantly higher than that of the normal population [64]. Multiple lines of evidence suggest that tau is transmitted between cells within an individual [65]. It was found that the intravenous injection of paired helical filaments (PHF)–tau protein from a human AD brain into 5×FAD transgenic mice could exacerbate neuroinflammation and Aβ and tau pathologies, showing increased p-tau immunoreactivity and increased deposits of Aβ_40_ and Aβ_42_ [66]. However, it has not been reported that tau spreads between animals through blood exchange and then causes neurofilament tangles. Based on the existing studies, it is just conjectured that tau might be infective through blood transmission.

#### 3.2.3. Adverse Events of Blood Transfusion

As a conventional technology for the treatment of clinical diseases, blood transfusion has been confirmed to be associated with some adverse events, such as iron overload [67], inflammation [68], and pathogen infection [69]. Blood transfusion brings extra iron to the recipients. The excess iron caused by repeated transfusion can cause iron metabolism disorders, leading to various serious complications such as damage to the liver, heart, nerves, and blood system [70,71]. The imbalance of iron homeostasis in the blood has adverse effects on the development and function of the nerves and may participate in the occurrence and development of AD [72]. Increased cortical iron may be strongly associated with neuro-cognitive dysfunction in prodromal AD [73]. Abnormal iron deposition was found in the cerebral cortex of patients with AD [74]. Besides that, iron depositions in the brains of patients with AD were obviously increased with magnetic resonance imaging sensitive to iron [75]. Combined with Aβ positron emission tomography, it was indicated that there was a positive correlation between iron deposition in the brain and cognitive impairment associated with Aβ deposition [76,77], which suggested that the deposition of iron and Aβ aggravated the impairment of cognitive function. Moreover, excess iron could also induce a variety of enzyme activities to promote tau phosphorylation [78]. After using iron chelators such as deferiprone and deferoxamine, the phosphorylation levels of tau in the brains of experimental animals decreased [79].

In addition, chronic inflammation has been shown to be closely related to cognitive impairment and the progression of AD [80], and can contribute to AD pathogenesis [68]. The particles from platelets, red blood cells, and white blood cells can trigger pro-inflammatory information during blood transfusion [81]. In a past study, the increase in inflammatory markers in the blood was related to the decline of cognitive function in the elderly [82,83], and the levels of many inflammatory cytokines such as interleukin-6, C-reactive protein, and α-1-antichymotrypsin were significantly increased in patients with AD [84].

In addition, herpesvirus can be transmitted by blood transfusion and then infect the recipients [85], and is intimately connected with the incidence of AD [69,86,87]. Herpesvirus can also initiate the amyloid cascade reaction, further promoting the fibrosis and deposition of Aβ [88,89]. Patients over 50 years old who were infected with herpes zoster virus had a significantly increased risk of developing AD, while those who received anti-herpetic therapy had a lower risk of AD [90]. More than 66% of people aged 0–49 worldwide carry the herpesvirus [91]. If infected with herpesvirus by blood transfusion, the recipient will have a significantly higher incidence of AD later in life. It must be pointed out that adverse events such as iron overload and chronic inflammation usually occur under the premise of repeated or massive blood transfusions. Therefore, considering these adverse events in a single low-volume transfusion is typically unnecessary.

**Figure 2:**
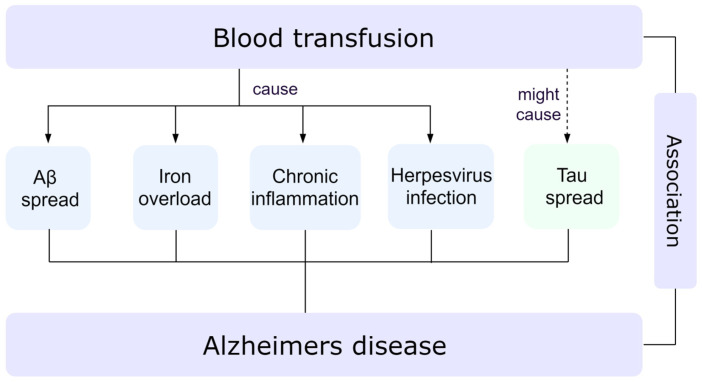
Possible mechanism of increasing risk of AD associated with blood transfusion.

Various approaches and mechanisms of the association between blood transfusion and AD were analyzed and summarized (Figure 2).

### 3.3. Epidemiological Studies on the Association of Blood Transfusion with the Risk of AD

The epidemiological studies on blood transfusion and the risk of AD were summarized (Table 2). Since the 1980s, several population-based, case–control studies have been performed to assess the potential risks of blood transfusion and AD [92,93,94,95,96]. These studies came to the same result, indicating that prior blood transfusion was not associated with an increased risk of AD. However, these studies had some limitations. For example, the sample size was small, or the long latency of AD was not adequately considered. Furthermore, given the prevalence of AD, the efficacy of case–control studies is limited. Additionally, the capacity of these studies to detect the subtle impact of transfusion history on AD risk warrants careful consideration [93].

Determining whether blood transfusion was associated with an increased risk of AD called for a large longitudinal cohort study. To investigate the association between neurodegenerative diseases and transfusion, Edgren et al. conducted a retrospective cohort study in 2016 on 1,465,845 patients who underwent blood transfusions from 1968 to 2012 [97]. The study utilized the Scandinavian Donations and Transfusions database for the long-term follow-up on donors and transfusion recipients, covering inpatient and outpatient care, cancer occurrence, emigration, and death [97]. Risk assessment was performed using disease concordance between the affected donors and their recipients, and disease incidence among all recipients of a particular donor [97]. For the first approach, receiving transfusions from donors who were later diagnosed with a neurodegenerative disease under investigation within two decades after donation was defined as exposure [97]. For the second approach, the cumulative time-dependent “disease excess score” for each donor was calculated and used as exposure [97]. Any type of dementia, AD, Parkinson’s disease, and amyotrophic lateral sclerosis were included as outcomes [97]. All transfusions during a 180-day exposure period; the recipient’s age, sex, and ABO blood type; the donors’ disease status; and other information were collected [97]. Follow-up began 180 days after the first recorded transfusion and continued until the date of first diagnosis of neurodegenerative disease, death, migration, or the end of follow-up (31 December 2012) [97]. Patients were excluded in the following three situations: (1) receiving an autologous unit or the inability to trace the donor’s identity; (2) receiving blood transfusions in areas without a completed patient registration in Sweden; and (3) death or diagnosis of any studied diseases within 180 days post-transfusion [97]. The hazard ratios for disease incidence in the recipients of blood from affected donors versus unaffected donors were evaluated using Cox regression, and the shared increased risk among multiple recipients of blood from the same donor was tested [97]. As a result, the study suggested that exposure to the blood of affected donors was not associated with the risk of AD (HR = 0.99; 95% CI, 0.85 to 1.15), and no disease concordance was found among multiple blood recipients of the same blood donor, indicating that such transmission was likely rare [97].

This study was characterized by a large number of samples, long periods of time, and abundant data. However, several limitations must be mentioned. Firstly, while it involved various neurodegenerative diseases, it was not specifically aimed at AD. Second, there was often a significant delay between AD onset and diagnosis, leading to an underestimation of the recipients exposed to risky blood who subsequently developed neurodegenerative diseases. Finally, this study focused solely on the risk for recipients of transfusions from donors with known neurodegenerative diseases and did not consider unknown donors or those without a history of blood transfusion.

In 2019, Lin et al. conducted a retrospective cohort study with the same objective in Taiwan, based on data from National Health Insurance Research Database (NHIRD), involving 63,813 patients who received one or more blood transfusions from 2000 to 2011 and 63,813 matched controls [98]. Having received blood transfusion was defined as exposure, and the main outcomes were dementia and AD. Basic information such as blood transfusion date and type, age, gender, and occupation, as well as the number of brain CT/MRI and psychiatric outpatient visits per year, the existence of diabetes, hypertension, obesity, anxiety, depression, post-traumatic stress disorder, bipolar disorder, schizophrenia, head injury, stroke, surgery history, chronic kidney disease, and other medical records and the history of using benzodiazepines, non-benzodiazepines, and antipsychotic drugs were collected and adjusted in multivariable Cox proportional hazards models [98]. The start of follow-up was delayed to 2 years after the transfusion date. Each participant was followed up until 31 December 2011, or until the main outcomes were diagnosed, or were censored due to death or withdrawal from the NHI program [98]. Patients with missing date of birth and gender data and those diagnosed with dementia or AD in the past and within 2 years after the start of the study were excluded.

Compared with the matched cohort, patients who received 1, 2, or ≥3 blood transfusions had a higher risk of developing dementia (*p* < 0.001) [98]. Surprisingly, although the risk of dementia persisted during the follow-up period, it decreased over time [98]. The highest risk of developing dementia occurred in the first 3 years of the follow-up, with a HR of 2.02 [98]. Afterwards, the risk decreased to 1.87 between 3 and 6 years of follow-up [98]. After 6 years of follow-up, the risk further decreased to 1.56 [98]. This trend may be attributed to two factors: firstly, frequent hospital visits by recipients could increase the likelihood of diagnosing dementia earlier; secondly, patients requiring transfusions may have a shorter life expectancy [98]. Multivariate Cox regression analysis showed that patients who received blood transfusions had a higher risk of developing AD than those who did not (HR = 1.37; 95% CI, 1.13 to 1.66) [98]. In addition, patients who received washed red blood cells had a higher risk of developing dementia than those who did not (HR = 2.37; 95% CI, 1.63 to 3.44) [98]. Unlike previous studies, this study suggested a correlation between blood transfusion and AD risk. However, since the family history information for each study subject was not available, this study could not evaluate the impact of genetic factors. Moreover, the follow-up time was short and there might not have been enough time to obtain the incidence of AD in young patients.

## 4. Discussion

The evidence regarding whether transfusions increase the risk of AD remains inconclusive. Two related retrospective cohort studies yield inconsistent results [97,98]. However, the research designs of the two studies differ, rendering their results incomparable. Edgren et al. defined exposure as receiving blood from a donor who developed a neurodegenerative disease within 20 years of donation, whereas Lin et al. focused on the act of receiving a blood transfusion itself. Additionally, the two studies controlled the confounding factors differently. Edgren et al. adjusted recipient age, gender, blood type, country, and donor disease status, while Lin et al. matched a considerable number of dementia-related comorbidities and medications. Furthermore, Edgren et al. conducted a follow-up period spanning 44 years, whereas Lin et al. initiated their first follow-up two years after the start of the study, with a total follow-up duration of nine years, eleven years after the initial transfusion.

However, the observed increase in the risk of AD just 11 years after exposure has prompted discussions regarding the incubation period of AD. Previous studies have reported that c-hGH injection-induced AD pathology typically has an incubation period of 30 to 40 years [47]. Two potential explanations for this discrepancy in latency are proposed. Firstly, AD occurrence may not relate to transfusion. Some AD cases may be sporadic, though the authors controlled for numerous confounding factors and still observed a significant difference in AD risk, indicating that sporadic cases alone cannot fully account for the observed phenomenon. Secondly, differences in population susceptibility may play a role. A notable distinction between the two groups lies in the age at exposure; most children receiving c-hGH infusion were approximately 10 years old, whereas the average age of the recipients in Lin’s study was 58.4. Similarly, Zhao et al. found an increased cumulative incidence of ICH within a decade of exposure, with a median age of 64 to 65 years at the start of the study [51]. In response, Zhao et al. hypothesized that differences in exposure age might influence the potential incubation period. This hypothesis is supported by the fact that age is a major risk factor for AD [99], and individuals in their 60s may already exhibit some degree of preclinical brain amyloidosis. Studies have demonstrated that Aβ oligomers can accelerate Aβ seeding [100]. Consequently, older individuals exposed to Aβ seeds may experience accelerated amyloidosis and shortened latency. Although this remains speculative, it provides valuable insights for future epidemiological studies of AD, such as stratifying by age or carefully setting appropriate follow-up durations.

In addition, Lin et al. found that transfusion was associated with the risk of dementia irrespective of transfusion frequency upon stratification [98]. However, their analysis indicated that the risk of dementia did not escalate with increasing transfusion frequency. This observation appears to contradict the intuitive expectation that a higher frequency of transfusions would correlate with an increased risk of dementia. Nonetheless, it would be premature to conclude that transfusion frequency is unrelated to dementia risk. Detecting subtle effects, such as the impact of the transfusion frequency on dementia risk, requires a more robust study design and greater statistical power. It is plausible that the methodology employed by Lin et al. may have lacked the requisite sensitivity to detect such nuanced associations.

Whether transfusions increase the risk of AD ultimately hinges on epidemiological and basic research. Additional prospective studies with stringent control and power should be conducted to demonstrate whether blood transfusion is associated with an increased risk of AD. Such studies would require establishing a large cohort of blood donors and recipients, similar to those in Sweden and Denmark. Exposure would be defined as receiving blood from donors who later developed AD. The primary outcome would be the occurrence of AD. It is indispensable to obtain the correlated neuropathologic findings through postmortem analysis. Since there is often a considerable delay between the onset and diagnosis of AD, long-term follow-up is necessary when conducting cohort studies.

A well-designed cohort study hinges on accurately determining exposure and outcomes, as well as controlling for confounding factors. Using the latest criteria revised by the National Institute on Aging and the Alzheimer’s Association to judge outcomes is recommended [101]. Amyloid positron emission tomography, approved cerebrospinal fluid biomarkers, and newly recognized accurate plasma biomarkers are defined as early-changing Core 1 biomarkers of AD, and an abnormal Core 1 biomarker result is sufficient to diagnose AD [101]. Using abnormal plasma biomarkers as a diagnostic criteria can provide exposure and outcome data more than ten years in advance [102], thereby shortening the research process and avoiding the underestimation of AD events and risks due to low diagnosis rates. Collecting plasma biomarker information at each follow-up and continuing to monitor donors can enhance the understanding of AD progression and evaluate the value of these biomarkers in early diagnosis.

Prospective cohort studies offer several advantages: they provide accurate data since both exposure and disease status are measured by investigators; they demonstrate a stronger causal link compared to case–control studies; and they allow dynamic observations to understand the natural history of AD. However, these studies also have limitations, including large sample sizes, high costs, long observation periods, and complex organizational requirements.

Alternative approaches include detailed case reports for abnormal early-onset AD patients, or case–control studies on early-onset AD patients, matching factors such as age, sex, region, AD genes, or APOE4 pathogenic mutations to explore the potential association between blood transfusion and early-onset AD cases. Future studies should be refined to differentiate the subtypes of AD and investigate their potential for transfusion transmission. In addition, some problems remain unclear, and the detailed mechanisms need to be further studied, including how Aβ in exogenous blood affects the recipients, whether tau causes AD pathology through blood transfusion, and what the association is between different frequencies, components, and volumes of blood transfusion and the risk of AD.

The possible transfusion transmission of AD has important public health implications and should promote clinical alertness relating to the safety of blood transfusion. At present, there is no effective method to eliminate proteopathic seeds in blood. Therefore, a more practical approach to mitigate the risk of potential transmission begins with blood donor screening. The World Health Organization (WHO) recommends the permanent deferral of blood donation for patients with dementia or any neurodegenerative diseases, individuals diagnosed with Creutzfeldt–Jakob disease, and those with a family history of this condition, as well as people who have received pituitary-derived human growth hormone, undergone dural transplantation, or had neurosurgery [103].

Should further research substantiate a potential risk of transmission, it may become necessary to include individuals with a familial predisposition to dementia or neurodegenerative diseases in the criteria for ineligibility. To prevent unnecessary exclusions, we also advocate for the assessment of plasma p-tau217 levels in such donors. This biomarker can serve as an effective indicator for identifying preclinical AD [101,104]. Additional safety measures might involve restricting transfusions from donors over a certain age to children or young adults [105]. The probability of herpesvirus infection is very high, and it is neither feasible nor useful to rule out past infections. In response, the WHO advises that symptomatic donors should postpone their donation until they have fully recovered [103].

Autotransfusion is also advocated, as it can avoid the infection of exogenous pathogens and the infusion of foreign biochemical substances. Unreasonable blood transfusion should be avoided, which can reduce the incidence of adverse events associated with transfusion. Blood transfusion services must perform well in blood screening and management. The health of those who receive blood from AD patients or people with a high risk factor for AD should be carefully monitored and followed up on, especially if the recipients are also AD patients. Furthermore, we recommend that blood banks, hospital transfusion departments, blood centers, and other relevant organizations collaborate effectively on registering and monitoring both blood donors and recipients. Concurrently, it is advisable to retain a small sample of blood from each donation bag for future follow-up studies.

## 5. Conclusions

Blood transfusion is a critical component of modern medicine. However, due to the complex nature of blood components, there exists a risk of disease transmission associated with this procedure. AD has emerged as a global concern, and its etiology is multifaceted. Although the precise pathogenesis remains to be fully elucidated, significant insights have been gained in understanding it. In the 1980s and 1990s, several case–control studies indicated that blood transfusion did not carry the risk of AD transmission. However, two retrospective cohort studies published in the past decade have yielded inconsistent results, with one suggesting an association between blood transfusion and an elevated risk of AD. The potential mechanism may involve Aβ, tau, iron overload, inflammation, and microbial infection. Certainly, we still need more systematic research on model organisms to ascertain whether blood transfusion raises the risk of AD. Furthermore, rigorous clinical data monitoring and the analysis of blood transfusion history alongside AD incidence are imperative.

## Figures and Tables

**Table 1:** Evidence of Aβ transmission between individuals.

Authors, Year	Species, Design, Exposure	Key Findings	Authors’ Conclusion
Gamez et al., 2022 [35]	Tg2576 mice. Animal research. Intra-lingual injections/extra-nasal administration of Aβ aggregates.	Intra-lingual delivery of Aβ aggregates quickens plaque emergence, while extra-nasal administration amplifies amyloid pathology in the olfactory bulb.	Retrograde axonal transport can carry Aβ seeds from peripheral areas to the brain.
Meyer-Luehmann et al., 2006 [36]	APP23 mice. Animal research. Intra-hippocampus injection of brain homogenates from AD patients or APP23 transgenic mice.	Cerebral β-amyloidosis and related pathologies were triggered in APP transgenic mice by intracerebral injection of dilute brain extracts containing Aβ.	Both the host and the agent’s source played a role in the phenotype of the induced amyloidosis.
Ridley et al., 2006 [37]	Marmosets. Animal research. Human or marmoset brain homogenate with Aβ was injected into the brain 1–8 years prior.	Most treated marmosets exhibited cerebral beta-amyloidosis.	In primates, cerebral amyloidosis can be triggered or accelerated by Aβ or its related factors.
Bu et al., 2018 [38]	APP/PS1 mice and their wild-type littermates. Animal research. Parabiosis was set up.	Formation of cerebral β-amyloidosis, tau phosphorylation, other AD-type pathologies, and impairment of hippocampal CA1 LTP were found in wild-type mice.	Blood-derived Aβ can enter the brain, causing Aβ-related pathologies and inducing neuronal functional defects.
Sun et al., 2021 [39]	APP/PS1 mice and wild-type mice. Animal research. Transplantation of bone marrow from APP/PS1 mice to wild-type mice.	Human Aβ was consistently expressed in the blood by bone marrow cells, causing AD phenotypes and behavioral deficits in the wild-type recipient mice one year after transplantation.	Aβ produced by blood cells plays an important role in the pathogenesis of AD.
Singh et al., 2024 [40]	AD mice, APP-knockout mice, and wild-type mice. Animal research. Bone marrow from AD mice was transplanted into both APP-knockout and wild-type recipients.	Pathological features of AD, such as impaired blood–brain barrier integrity, elevated brain Aβ levels, and cognitive impairment, were found within 6–9 months after transplantation.	Donor stem cells carrying a pathogenic mutant allele can effectively transmit central nervous system disorders to healthy recipients.
Burwinkel et al., 2018 [41]	APP/PS1 mice. Animal research. Intravenous injection of brain extracts from AD patients once.	Vascular Aβ amyloidosis was promoted in APP/PS1 mice that received the injection.	In APP/PS1 mice, the intravenous administration of human Aβ seeds facilitated the deposition of Aβ in blood vessels.
Morales et al., 2020 [42]	Tg2576 and APP/PS1 mice. Animal research. Blood from old transgenic mice was injected into young transgenic mice.	Young transgenic mice exhibited notably elevated levels of brain amyloidosis and neuroinflammation.	Aβ seeds in the blood can enter the brain, promoting neuropathological changes in the recipient mice.
Ritchie et al., 2017 [43]	Human. Comprehensive study. hGH-iCJD.	Compared with age-matched patients who died of sCJD and vCJD, patients with hGH-iCJD had a higher incidence of Aβ accumulation in the brain and cerebral blood vessels.	Aβ seeding can occur in the human brain without abnormal prion proteins.
Frontzek et al., 2016 [44]	Human. Autopsy study. Succumbed to iCJD after dural grafting.	Compared with sCJD patients, iCJD patients had a higher risk of congophilic amyloid angiopathy and amyloid plaque deposition in the brain.	Aβ deposition is commonly observed in the brains of iCJD patients following dura mater transplantation, leading to congophilic amyloid angiopathy and the formation of parenchymal plaques.
Jaunmuktane et al., 2015 [45]	Human. Autopsy study. ICJD after c-hGH therapy.	In four of eight iCJD patients, both parenchymal and vascular Aβ deposition were observed, consistent with the hypothesis of iatrogenic transmission of Aβ pathology.	Healthy exposed individuals may also have the risk of iatrogenic AD and CAA.
Purro et al., 2018 [46]	In vitro research.	Aβ seeds and tau proteins were identified in the archived c-hGH vials, and the preserved c-hGH could induce Aβ deposition and CAA in *App*^NL-F/NL-F^ mice.	These findings support the hypothesis that injection of c-hGH can lead to the transmission of Aβ pathology.
Banerjee et al., 2024 [47]	Human. Case series. Received treatment with c-hGH prepared by Wilhelmi preparation technique.	Five of eight developed AD approximately three decades later. Iatrogenic AD differed phenotypically from patients with sporadic and familial AD.	AD has three forms: iatrogenic, sporadic, and hereditary.
Raposo et al., 2020 [48]	Human. Case report. Underwent cardiac surgery involving a cadaveric dura mater patch at 2 years old.	Early-onset CAA occurred three decades after the surgery and manifested as multiple ICHs.	Aβ seeds may enter the host via blood from the cardiac dural patch and further spread to the brain.
Banerjee et al., 2019 [49]	Human. Case series. Received neurosurgery or neurovascular surgery using cadaveric dura mater materials during childhood.	Three patients developed early onset CAA decades after childhood exposure to cadaveric dura.	Aβ seeds found in the cadaveric dura could potentially be transmitted through surgery, leading to CAA in the recipient.
Jaunmuktane et al., 2018 [50]	Human. Case series. Underwent neurosurgical procedures during childhood or teenage years.	Four patients presented with ICH caused by severe CAA approximately three decades after neurosurgical procedures.	The spread of Aβ seeds may be caused by using surgical instruments carrying misfolded Aβ.
Zhao et al., 2023 [51]	Human. Cohort study. Received red blood cell transfusions from donors who subsequently experienced multiple spontaneous ICHs.	Individuals who underwent transfusions from donors who subsequently experienced multiple spontaneous ICHs had a higher risk of spontaneous ICH.	Transfusion-transmission agents may be associated with some types of spontaneous ICHs.

Abbreviations: Aβ, amyloid-β; AD, Alzheimer’s disease; c-hGH, cadaveric-derived human growth hormone; CAA, cerebral amyloid angiopathy; CJD, Creutzfeldt–Jakob disease; iCJD, iatrogenic Creutzfeldt–Jakob disease; sCJD, sporadic Creutzfeldt–Jakob disease; vCJD, variant Creutzfeldt–Jakob disease; and ICHs, intracerebral hemorrhages.

**Table 2:** Epidemiological studies on blood transfusion and Alzheimer’s disease risk.

Author, Year	Study Design/Type, Objective	(No. of Participants) Exposure/Case Group vs. (No. of Participants) Control Group	Main Findings	Authors’ Conclusion
Heyman et al., 1984 [94]	Case–control study. To determine the possible roles of various factors in the development of AD.	(40) Patients with dementia before age 70. vs. (80) Age-, gender-, and race-matched community control subjects.	Significantly higher frequency of prior thyroid disease and history of severe head injury were found in female patients than in female control subjects. No other significant differences were found.	Apart from a history of thyroid disease and severe head injury, no other demographic or clinical factors related to AD were observed.
Amaducci et al., 1986 [95]	Case–control study. To identify possible risk factors for clinically diagnosed AD.	(116) Patients with a clinical diagnosis of AD in seven Italian centers. vs. Age-, sex-, and residence-matched hospital controls (160) and population controls (97).	The association between blood transfusion and AD was not found (hospital: OR = 0.73; population: OR = 0.71).	AD is associated with a family history of dementia and maternal age at delivery.
Broe et al., 1990 [96]	Case–control study. To identify possible risk factors for AD.	(170) Cases aged 52 to 96 years who were clinically diagnosed with AD. vs. (170) Controls matched for age, sex, and, as far as possible, general birth habits.	The association between blood transfusion and AD was not found (OR = 0.63).	Dementia history, possible AD, Down’s syndrome in first-degree relatives, and lack of exercise are risk factors for AD.
Bohnen et al., 1994 [92]	Case–control study. To assess whether blood transfusion is a risk factor for AD.	(252) AD cases who had lived in Olmsted County for 40 years or more. vs. (252) Age- and gender-matched controls.	Exposure to at least one blood transfusion, three or more transfusions, or at least six blood transfusions was not associated with an increased risk of AD.	Blood transfusion was not found to significantly increase the risk of AD.
O’Meara et al., 1997 [93]	Case–control study. To evaluate the possibility of blood-borne transmission of AD.	(326) Newly confirmed probable AD cases. vs. (330) Age- and sex-matched control subjects from the same healthcare organization.	The frequency of blood transfusion in the control group was higher than that in the case group (crude OR = 0.62). Stratified analysis by the APOE-e4 genotype showed no effect modification.	Whether or not APOE-e4 status was considered, transfusion history was not associated with an increased risk of AD.
Edgren et al., 2016 [97]	Retrospective cohort study. To investigate possible transfusion transmission of neurodegenerative disorders.	(42,254) Who had received transfusions from donors who were subsequently diagnosed with a neurodegenerative disease within two decades after donation. vs. (1,423,591) Received transfusions from donors who were not diagnosed with any neurodegenerative disorder within 20 years after donation.	The HR of AD in people receiving blood from dementia donors and healthy donors was 0.99.	There is no evidence to support transfusion-transmitted neurodegenerative diseases.
Lin et al., 2019 [98]	Retrospective cohort study. To investigate the association between neurodegenerative diseases and transfusion.	(63,813) Aged ≥ 20 years who underwent a blood transfusion. vs. (63,813) Control subjects with matched propensity scores who had no history of blood transfusion at baseline.	Compared with the control group, the HRs of dementia and AD in the transfusion group were 1.73 and 1.37, respectively.	Blood transfusion is associated with an increased risk of dementia and AD.

Abbreviations: AD, Alzheimer’s disease; HR, hazard ratio; and OR, odds ratio.

## Data Availability

No new data were created or analyzed in this study. Data sharing is not applicable to this article.
